# Unraveling the role of the circadian clock genes in cervical squamous cell carcinoma and endocervical adenocarcinoma: A prognostic indicator for prognostic, immunotherapy response, and chemotherapy sensitivity

**DOI:** 10.7150/jca.94063

**Published:** 2024-03-19

**Authors:** Jiayu Xu, Xueyuan Huang, Siqi Gou, Huanyu Luo, Shicheng Zeng, Qinhong Zhang, Qibiao Wu, Hao Chi, Guanhu Yang

**Affiliations:** 1 Faculty of Chinese Medicine, State Key Laboratory of Quality Research in Chinese Medicine, and University Hospital, Macau University of Science and Technology, Macao, Macao SAR, China.; 2 School of Science, Minzu University of China, Beijing, China.; 3 School of Clinical Medicine, Affiliated Hospital of Southwest Medical University, Luzhou, China.; 4 Children's Hospital of Chongqing Medical University, Chongqing, China.; 5 Heilongjiang University of Chinese Medicine, Harbin, China.; 6 Department of Specialty Medicine, Ohio University, Athens, OH, United States.

**Keywords:** cervical squamous cell carcinoma and endocervical adenocarcinoma, circadian clock genes, tumor microenvironment, prognostic markers, immunotherapy response, chemotherapy sensitivity

## Abstract

**Background:** Cervical squamous cell carcinoma and endocervical adenocarcinoma (CESC) account for a significant proportion of gynecological malignancies and represent a major global health concern. Globally, CESC is ranked as the fourth most common cancer among women. Conventional treatment of this disease has a less favorable prognosis for most patients. However, the discovery of early molecular biomarkers is therefore important for the diagnosis of CESC, as well as for slowing down their progression process.

**Methods:** To identify differentially expressed genes strongly associated with prognosis, univariate Cox proportional hazard analysis and least absolute shrinkage and selection operator (LASSO) regression analysis were used. Using multiple Cox proportional hazard regression, a multifactorial model for prognostic risk assessment was then created.

**Results:** The expression of biological clock-related genes, which varied considerably among distinct subtypes and were associated with significantly diverse prognoses, was used to categorize CESC patients. These findings demonstrate how the nomogram developed based on the 7-CRGs signature may assist physicians in creating more precise, accurate, and successful treatment plans that can aid CESC patients at 1, 3, and 5 years.

**Conclusions:** By using machine learning techniques, we thoroughly investigated the impact of CRGs on the prognosis of CESC patients in this study. By creating a unique nomogram, we were able to accurately predict patient prognosis. At the same time, we showed new perspectives on the development of CESC and its treatment by analyzing the associations of the prognostic model with immunity, enrichment pathways, chemotherapy sensitivity, and so on. This research provides a new direction for clinical treatment.

## Introduction

Cervical squamous cell carcinoma (SCC) and endocervical adenocarcinoma (EAC) are the two major histological subtypes of cervical cancer and are collectively known as CESC. CESC accounts for a significant proportion of gynecological malignancies and represents a major global health concern. The development of these subtypes is primarily associated with persistent infection by high-risk human papillomavirus (HPV). As the infection is usually asymptomatic 1, it can take 10 to 15 years for changes in the cervix to become apparent 2. According to statistics from 2018, cervical cancer is the second most prevalent cancer in women worldwide and the fourth most common cancer overall among women in nations with poor human development indices. It is crucial to identify early molecular indicators to diagnose CESC and to stop its progression. Although the different etiologies and clinical behaviors of CESC have been well studied 3-6, the specific underlying molecular processes are not entirely understood.

The human body exhibits 24-hour rhythmic variations in its physiological processes and behaviors, which are governed by an autonomous biological pacemaker called the circadian clock 7. Disruptions in these rhythms have been linked to the initiation and progression of cancer 8-11. Epidemiological investigations of individuals working night shifts have revealed the critical role of circadian rhythm disturbances in the development of breast cancer 12, skin cancer 13, colorectal cancer 14, prostate cancer 15, and endometrial cancer 16. The CLOCK and BMAL1 proteins form heterodimers and activate the expression of CRY and PER genes 17, which, upon translation, inhibit the activity of CLOCK/BMAL1 and consequently repress their transcription 7. The levels of BMAL1 are further modulated by the transcription factors REV-ERBA/b 18 and RORA(1) 19, resulting in negative and positive regulation, respectively. Circadian clock mechanisms and clock gene expression are present in all nucleated cells 20. Remarkably, approximately 40% of the genome undergoes circadian regulation 21, underscoring the critical role of the circadian clock in cellular biology 22, 23. Dysregulation of this system has been implicated in various disease states, including cancer 24-28. Although circadian disruptions have been elucidated in certain cancer types, the relationship between clock genes and the CESC has yet to be definitively established 29-32. To date, there is a paucity of literature documenting the mechanisms underlying the circadian rhythm in cervical cancer, a concern given the increasing prevalence of these malignancies in developing countries, necessitating further scientific inquiry.

Therefore, in this study, we aimed to investigate the expression of circadian clock genes in CESC, with the overarching objective of unraveling the potential interplay between circadian rhythm alterations and the pathogenesis of CESC. Moreover, we sought to evaluate the efficacy of these genes as biomarkers for prognosticating disease severity and treatment outcomes. Our findings unveiled a downregulation of multiple clock genes in cervical cancer cells. Nonetheless, it is noteworthy that these cells exhibited preserved functional circadian oscillations. Additionally, our study demonstrated the inhibitory effects on cancer biology stemming from interference with clock gene expression activity. Dysregulation of clock genes in cancer cells has been postulated to be an attractive avenue for therapeutic intervention, as these genes are considered "druggable" targets 23. Collectively, our research substantiates the necessity for further investigation into the potential utility of the circadian clock as an innovative anticancer strategy.

## Methods

### Patient data source

From the tumor-associated gene database (https://portal.gdc.cancer.gov/), we were able to gather clinical information on 307 tumor patients as well as tumor-related gene expression patterns. TPM_n_=FPKM_n_*10^6^/(FPKM_0_+...+FPKM_m_), where n stands for gene n and m for the total number of genes, was used to convert the third level HTSeq-Fragments per kilobase of transcript per million mapped reads (FPKM) data from TCGA-CESC to transcripts per million (TPM). The TPM readings were then subjected to a log^2^-based modification. The GSE44001 database's gene profiles and clinical information for 300 CESC patients were retrieved from the GEO database (https://www.ncbi.nlm.nih.gov/geo/). The dataset used for external validation was GSE44001.

### Differential gene expression analysis

The "limma" tool in R was used to perform correction, normalization, and log2 transformation on the three original datasets. The GeneCards website (https://www.genecards.org/) provided us with a total of 1471 CRGs. Differential levels of CRGs expression were discovered. All the samples were divided into various clusters 33 using the "ConensusClusterPlus" method to further clarify the features of CRGs in CESC. The "pheatmap" tool in R was used to visualize the differential expression of CRGs and clinicopathological markers within various clusters. The "c2.cp.kegg.v7.5.1.symbols" gene set, which was obtained from the MSigDB database, was used for gene set variation analysis (GSVA). The "GSVA" approach was used to examine the variations in pathways between clusters 34. The ssGSEA algorithm 35 was used to perform immune cell infiltration analysis and immune checkpoint expression analysis on several clusters.

### Model construction and validation

We identified 33 genes linked to survival using univariate Cox regression analysis. The penalty regularization parameter lambda was then determined by LASSO regression analysis using the "glmnet" R package 36. We discovered a group of seven genes as a result of our investigation. The coefficients of the core genes were then determined and calculated using a stepwise multivariate Cox regression model. A risk signature for the CRGs was subsequently created. The CRGs risk score for each patient was determined as follows: Expression mRNA_1_CoefmRNA_1_+Expression mRNA_2_CoefmRNA_2_+...Expression mRNA_n_CoefmRNA_n_ are the components of the risk score.

### Independent predictive analysis and Nomogram construction

Based on the obtained model equation, risk scores were produced for all CESC patients, and the median was estimated using the "Survminer" package in R. The CESC patients were then split into low-risk and high-risk groups, and survival curves for each grouping were drawn. To evaluate the genetic signature's prediction power, the C-index was calculated in R using the "pec" package. Utilizing the "Time-ROC" R package, receiver operating characteristic (ROC) curve analysis was done to assess the genetic signature's ability to predict outcomes.

The risk score was evaluated as an independent prognostic factor using univariate and multivariate Cox regression models. Histograms were created using the "RMS" R package to estimate the survival of patients in the TCGA-CESC cohort based on the risk score and clinicopathological parameters.

### Functional concentration analysis

To investigate the functional annotations and enriched pathways of differentially expressed genes linked to CRGs in CESC, functional enrichment analysis was conducted. The "ClusterProfiler" R program was used to analyze the Gene Ontology (GO) pathways, and a significance threshold of P 0.05 was used to denote statistical significance. Using the "c2.cp.kegg.v7.5.1.symbols.gmt" dataset from MSigDB, the "GSVA" R package was used to perform GSVA. Heatmaps were produced using the "hotmap" R package. For subgroup comparisons, adjusted P-values of 0.05 based on the "limma" R program were regarded as statistically significant.

### Immunological analysis of risk characteristics

A variety of methods, including XCELL 37, 38, TIMER 39, 40, QUANTISEQ 40, 41, MCPCOUNT 41, EPIC 42, CIBERSORT 43, and CIBERSORT-abs 44, have been employed for quantifying immune infiltration scores. The link between immune cells and risk ratings was evaluated using Spearman correlation analysis. Based on the immune cell characteristics of people with CESC, the ssGSEA technique was used to distinguish between low-risk and high-risk patients. A prepared list of 20 immune checkpoint inhibitors taken from Auslander et al. 45 was used to compare immune checkpoint inhibition between high-risk and low-risk groups. The "estimate" R program was used to calculate immunological and stromal scores from RNA-seq data to assess tumor purity. The "limma" and "ggpubr" R packages were used to measure and visualize the immune treatment response in CESC patients. The "ggcor" R program was used to examine the relationship between risk scores and these two genetic features.

### Drug sensitivity

Based on the half-maximal inhibitory concentration (IC50) from the Genomics of Drug Sensitivity in Cancer (GDSC) dataset (https://www.cancerrxgene.org) 46, the "prediction" R package was used to evaluate the treatment response of patients in the high-risk and low-risk groups.

### TISCH analysis

A single-cell RNA sequencing database on the tumor microenvironment (TME) was made available at the Tumor Immune Single Cell Hub (TISCH, http://tisch.comp-genomics.org), which also offered thorough single-cell level cell type annotations. This made it possible to examine gene expression in particular cell types in more detail. The TME variations among different CESC patients were discovered by looking at the expression of cell type-specific genes in various cell types, partially explaining the variety of CESC.

### Statistical analysis

Statistical analysis was performed using R software version 4.2.1. Kaplan-Meier (KM) survival curves and log-rank tests were used to compare the overall survival (OS) of the high-risk and low-risk groups. The use of LASSO regression analysis was used to choose the candidate CRGs. Then, to create the CRGs features, a stepwise multivariate Cox regression analysis was performed. Using time-dependent ROC analysis, the model's prediction abilities were assessed. Using Spearman correlation analysis, the relationship between risk score and immune cell infiltration was evaluated. The Wilcoxon test was used to assess the proportion of tumor immune infiltrating cells (TIICs), immunological checkpoints, and immune function between the two groups. A p-value < 0.05 was considered statistically significant, and an FDR (false discovery rate) < 0.05 was also considered statistically significant.

## Results

### Identification of candidate CRGs and identification of molecular subtypes of CRGs by consensus clustering

**Figure 1:**
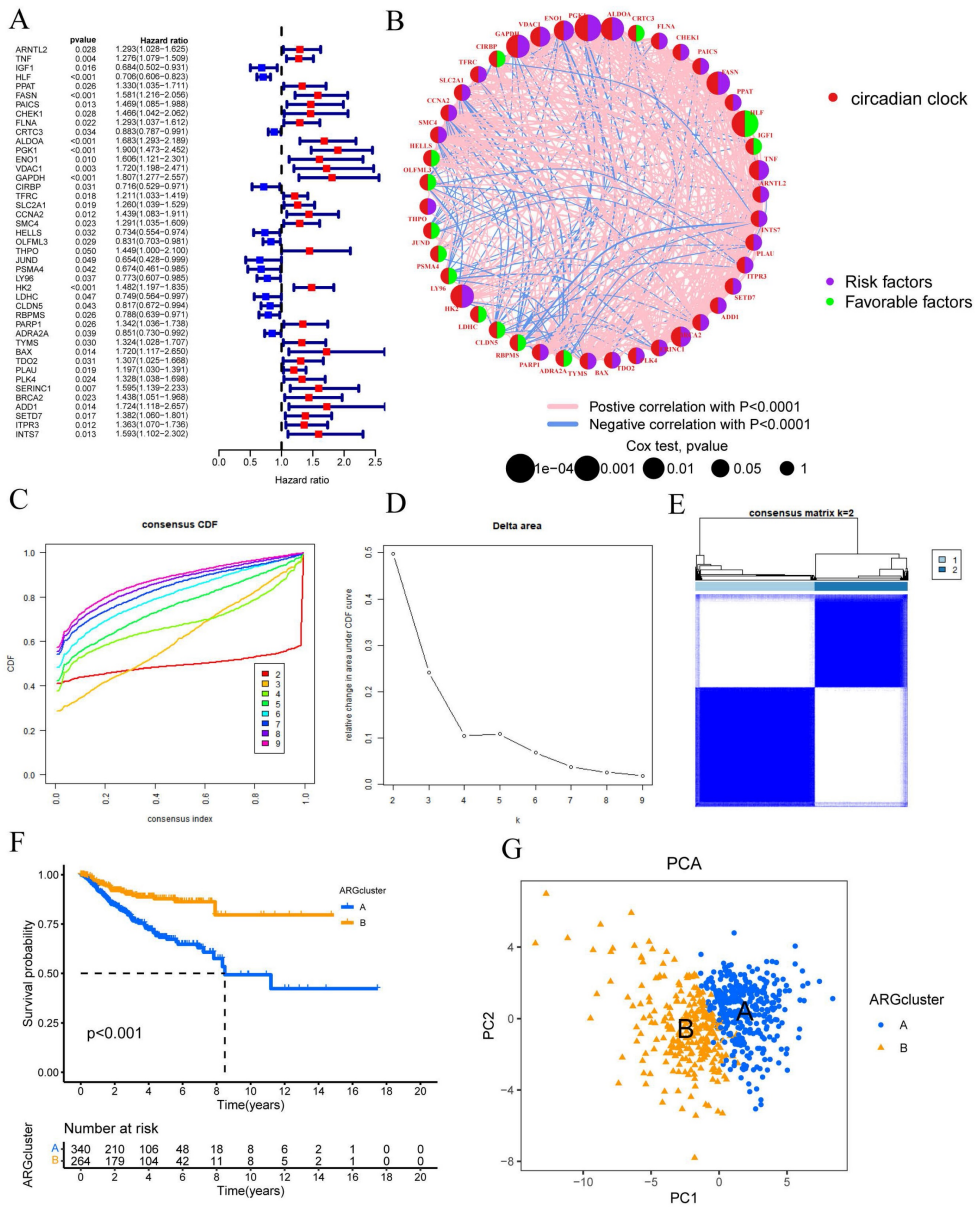
Univariate Cox regression analysis was conducted to assess the prognosis of 43 CRGs in the CESC cohort. The molecular subtypes of CRGs were determined using the consensus clustering method. (A) Prognostic analysis of the 43 CRGs. (B) Correlation analysis of the prognostic outcomes associated with the 43 CRGs. (C) Cumulative distribution function (CDF) of the consensus clustering when k ranged from 2 to 9. (D) Relative changes in the area under the cumulative CDF curve as k varied from 2 to 9. (E) Consensus matrix at k=2. (F) Differential overall survival (OS) between Cluster A and Cluster B. (G) Principal component analysis plot between Cluster A and Cluster B.

Using the "limma" package in R, a univariate Cox regression analysis was performed on 1471 differentially expressed CRGs to identify 43 CRGs associated with OS (P<0.05) (FIGURE 1A). Subsequent prognostic analysis was conducted on these 43 CRGs (FIGURE 1B).

Clustering was suggested by the cumulative distribution function's (CDF) rising tendency as compared to the consensus index. The CDF curve and delta area were used to estimate the best point for creating unique clusters, which was found to be k=2. The disparities between groups were greatest as the clustering index "k" rose from 2 to 9. To categorize CESC patients, two subgroups were created (FIGURE 1C-D). The consensus matrix also provided a better visualization tool to evaluate the composition and quantity of clusters. When k=2, we created a heatmap with colors that matched the consensus matrix. This heatmap showed high intra-group correlation and low inter-group correlation. This provided compelling evidence that categorizing CESC patients into Cluster A and Cluster B subtypes (FIGURE 1E) was appropriate. Patients in Cluster A were shown to have a better chance of surviving than those in Cluster B (P 0.001) (FIGURE 1E). Risk distribution in various populations is frequently visualized using PCA. When depicted using alternative clusters, patients from Cluster A and Cluster B revealed appreciable differences (FIGURE 1G).

### Molecular subtype enrichment and immunoassay for CRGs

**Figure 2:**
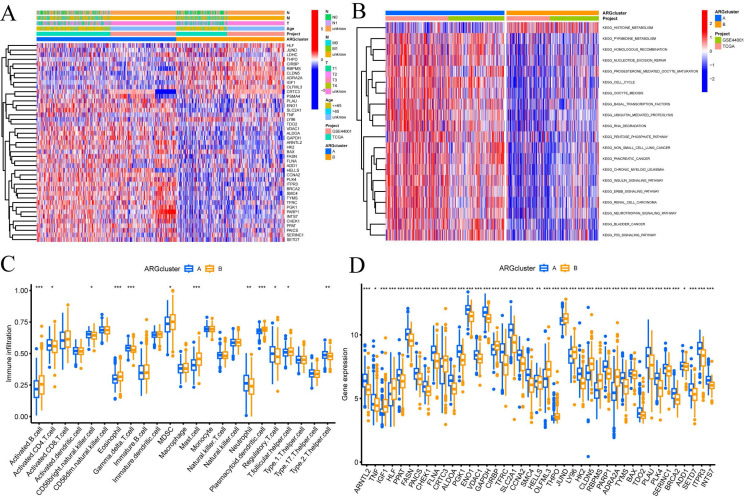
Enrichment and immune analysis of CRG molecular subtypes. (A) Associations between CRG expression and clinicopathological parameters. (B) KEGG enrichment analysis of different Clusters. (C) Immune cell scores comparison between Cluster A and Cluster B. (D) Differential expression of immune checkpoints between Cluster A and Cluster B. * P < 0.05, ** P < 0.01, *** P < 0.001, ns>0.05.

In addition, we conducted further investigations into the metabolic disparities among Cluster A, Cluster B, and CRGs. The utilization of a heatmap unveiled that Cluster A demonstrated greater variations in expression and clinical attributes within the CRGs (FIGURE 2A). To unravel the underlying biological pathways, we conducted enrichment analysis on distinct cluster samples employing the Kyoto Encyclopedia of Genes and Genomes (KEGG) pathway database. This analysis established connections with diverse pathways linked to cancer, including HISTIDINE METABOLISM, BASAI TRANSCRIPTION FACTORS, CELL CYCLE, and NUCLEOTIDE EXCISION REPAIR (FIGURE 2B).

Considering the indispensable role of immune therapy in cancer treatment, our focus was to comprehend the distribution and correlations of 23 TIICs in this cohort. By employing the ssGSEA algorithm, we accurately determined the levels of immune cell infiltration and subsequently classified them into distinct clusters. The outcomes revealed higher levels of infiltration in immune cells such as Activated B cells, Activated CD4 T cells, Eosinophils, MDSCs, Mast cells, Plasmacytoid dendritic cells, and T follicular helper cells within Cluster B as compared to Cluster A. Conversely, CD56bright natural killer cells, Gamma delta T cells, Neutrophils, Regulatory T cells, and Type 2 T helper cells exhibited lower levels of infiltration in Cluster B (FIGURE 2C).

These findings empower the CRGs risk-scoring model, enabling the categorization of diverse immune subtypes, which, in turn, have the potential to influence the response to immune therapy. Furthermore, considering the profound significance of immune checkpoints in the effectiveness of tumor immunotherapy and their pivotal role as a hallmark of the TME, we investigated the differential expression of immune checkpoint genes between the two clusters. Ultimately, we observed a substantial upregulation in the expression of immune checkpoint genes in patients belonging to Cluster A. Based on these comprehensive analyses, we concluded that Cluster A and Cluster B exhibit distinct expression profiles in the immune milieu, with Cluster A showcasing heightened effectiveness and sensitivity toward immune therapy (FIGURE 2D).

### Construction and validation of CRG signatures

**Figure 3:**
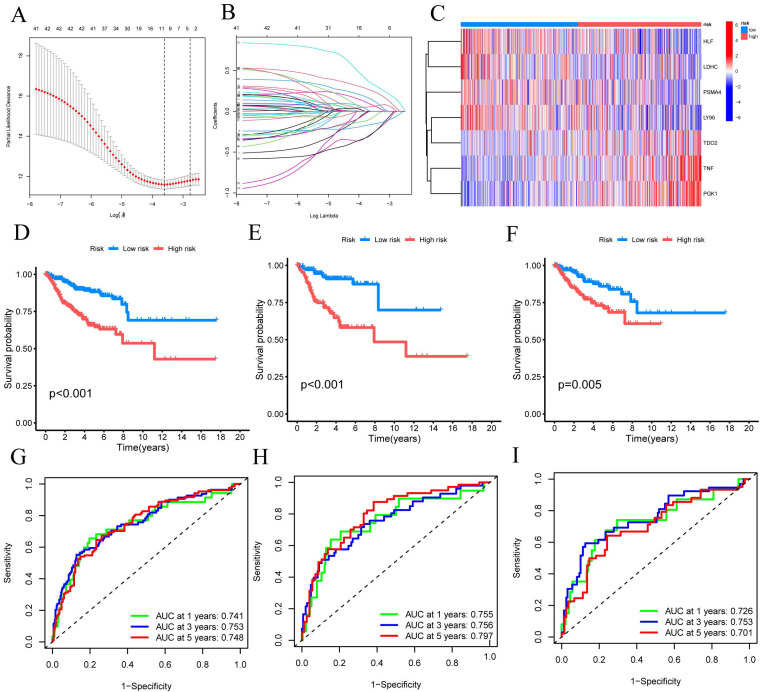
Building and verifying the 7-CRGs Signature. (A) Ten-fold cross-validation for choosing LASSO model tuning parameters. (B) profiles of LASSO coefficients. The risk factors in high-risk and low-risk patients are shown in a heatmap in (C). Kaplan-Meier curves (D) overall, (E) training set, and (F) validation set displaying the status of low-risk and high-risk UVM patients in the TCGA cohort. Time-dependent ROC curves for OS at 1 year, 3 years, and 5 years (G) overall, (H) training set, and (I) validation set.

Using univariate Cox regression analysis, we have identified a total of 33 CRGs that exhibit significant associations with OS in patients diagnosed with CESC. For candidate CRG identification, the LASSO machine learning method was implemented. Through the utilization of LASSO regression analysis, we meticulously selected nine candidate model genes, subsequently employing multifactor Cox proportional hazards regression modeling to further refine the data's dimensionality, ultimately narrowing down the selection to seven CRGs (FIGURE 3A-B). These seven CRGs, namely TNF, HLF, PGK1, PSMA4, LY96, LDHC, and TDO2, have been conclusively established as independent prognostic factors. The prognostic index (PI) was computed as follows: (PI) = (0.388 *TNF exp) + (-0.254 *HLF exp) + (0.595 *PGK1 exp) + (-0.528 *PSMA4 exp) + (-0.574 *LY96 exp) + (-0.369 *LDHC exp) + (0.322*TDO2 exp). Moreover, prognostic risk scores were computed for each patient, and based on the median score, CESC patients were dichotomized into high-risk and low-risk groups. The distribution of these seven CRGs within the high-risk and low-risk groups was illustrated, with HLF, LDHC, PSMA4, and LY96 exhibiting diminished expression levels in the high-risk group, while TDO2, TNF, and PGK1 displayed elevated expression levels among high-risk patients (FIGURE 3C).

Subsequently, these findings were employed to conduct survival analysis, thus corroborating their prognostic value. In the TCGA-CESC cohort, the mortality rate of all CESC patients exhibited an increase commensurate with elevated risk, whereas the low-risk group demonstrated a more favorable prognosis (P<0.001) (FIGURE 3D). To evaluate the efficacy of the created model for predicting OS in CESC patients, time-dependent ROC curves were used. AUC values of 0.741 at 1 year, 0.753 at 3 years, and 0.748 at 5 years were shown by the full time-dependent ROC curve (FIGURE 3G). In the training set, the mortality rate for CESC patients showed an increase consistent with higher risk (FIGURE 3E). Patients with low risk in the training set had a better prognosis than patients with high risk, according to KM survival analysis (P = 0.005). The AUC on the time-dependent ROC curve was 0.755 after one year, 0.756 after three years, and 0.797 after five years (FIGURE 3H). Patients with low risk showed a superior prognosis compared to patients with high risk in the testing set, according to KM survival analysis of CESC patients (FIGURE 3F). For the testing set, the time-dependent ROC curve showed AUC values of 0.726 at 1 year, 0.753 at 3 years, and 0.701 at 5 years (FIGURE 3I). We can conclude that our prognostic model effectively displays higher performance in light of these results.

### Constructing a column plot incorporating clinical features

**Figure 4:**
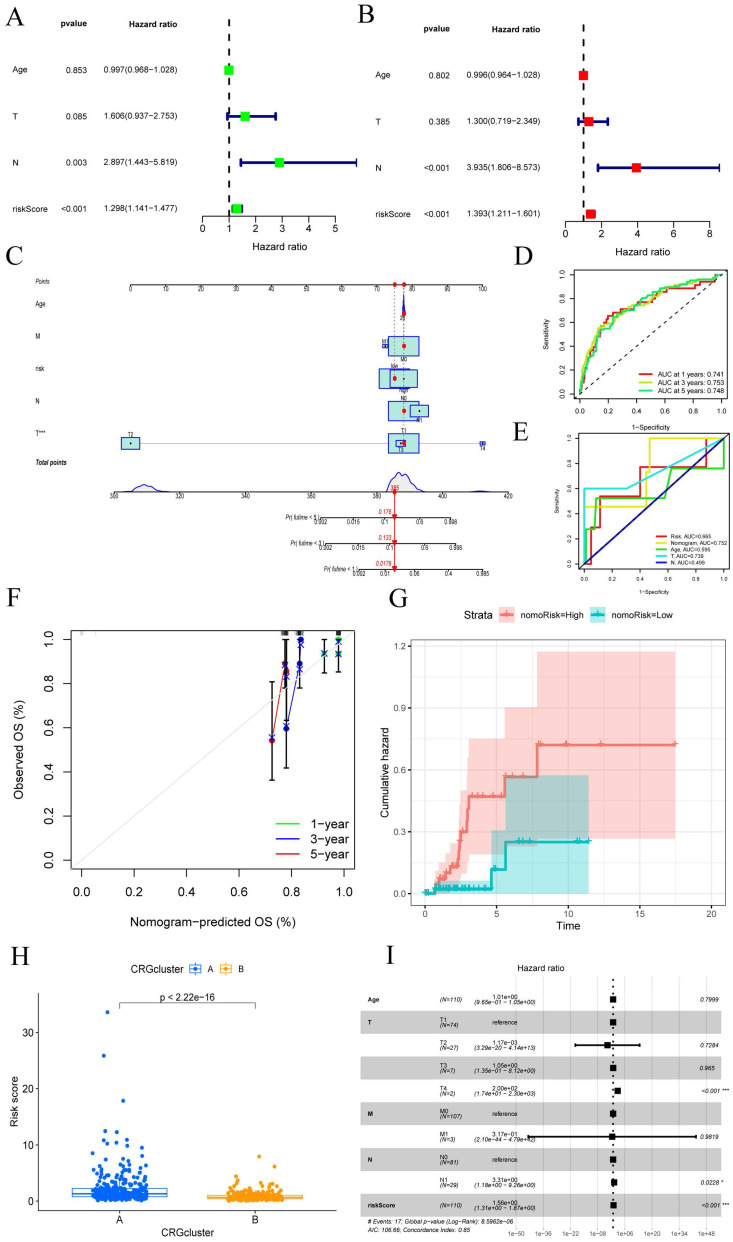
CESC created a nomogram by combining clinical features. (A-B) Analysis of the signature and distinct clinical features using univariate and multivariate COX regression. Univariate Cox analysis of the HR values for each variable is shown in green squares. Multivariate Cox analysis of the HR values for each variable is shown in red squares. (C) A nomogram made up of CRGs, Age, T stage, and N stage is an option. (D) Analysis of time-dependent ROC curves. (E) The time-dependent ROC curves for the nomogram. (F)The generated nomogram of 1-year, 3-year, and 5-year survival calibration curves. (G) The cumulative hazard curve showed how the likelihood of survival changed over time. (H) Prognostic variations in risk scores according to subtype. (I) A summary of the multivariable Cox regression analysis of the clinical characteristics and risk score in CESC patients using a forest plot.

Given the robust association observed between the constructed risk model and unfavorable prognosis, we conducted univariate and multivariate Cox analyses integrating the OS data of CESC patients alongside their clinical characteristics. We aimed to investigate whether the prognostic features derived from the 7-CRGs could function as independent prognostic predictors. Univariate analysis incorporating variables such as age, T stage, N stage, and risk score unveiled a notable correlation between the risk score and the prognosis of CESC patients (P<0.001) (FIGURE 4A). Remarkably, even after performing multivariate analysis, the risk score persisted as the most robust and autonomous prognostic factor in the cohort (P<0.001) (FIGURE 4B). We developed a nomogram incorporating age, T stage, N stage, and risk score as prognostic indicators for 1-year, 3-year, and 5-year survival probability in CESC patients to increase the clinical application and usability of the constructed risk model (FIGURE 4C). The results highlighted the risk model based on the 7-CRGs' improved prognostic capability for predicting the course of CESC patients. Furthermore, the 1-year, 3-year, and 5-year OS probability calibration curves showed a more positive concordance between projected and actual values (FIGURE 4D). Additionally, (FIGURE 4E) comparison of the column plot, risk, and common clinicopathological feature column plots showed that the 7-CRGs-based risk model had greater predictive power compared to models that only used age, T stage, N stage, and risk score (AUC=0.752). This supports the strong predictive ability displayed by our developed 7-CRGs characteristics when compared to other clinical parameters. Further evidence of good agreement between projected and observed probabilities of 1-year, 3-year, and 5-year OS was provided by the calibration curves (FIGURE 4F). Additionally, the column plot's cumulative risk curve showed that the risk of overall survival rose gradually for CESC patients with higher scores (FIGURE 4G). Additionally, the analysis of risk scores revealed a significant divergence in prognosis between CRGs cluster A and cluster B (p < 2.22e-16) (FIGURE 4H), providing further corroboration of the commendable properties of our model. Subsequently, the forest plot displayed within the nomogram underscored the prominent influence of risk score and T stage as major contributing factors (FIGURE 4I). These findings collectively endorse the column plot of risk scores derived from the 7-CRGs as an effective approach for prognostic prediction in clinical practice.

### Functional enrichment analysis of CRGs

**Figure 5:**
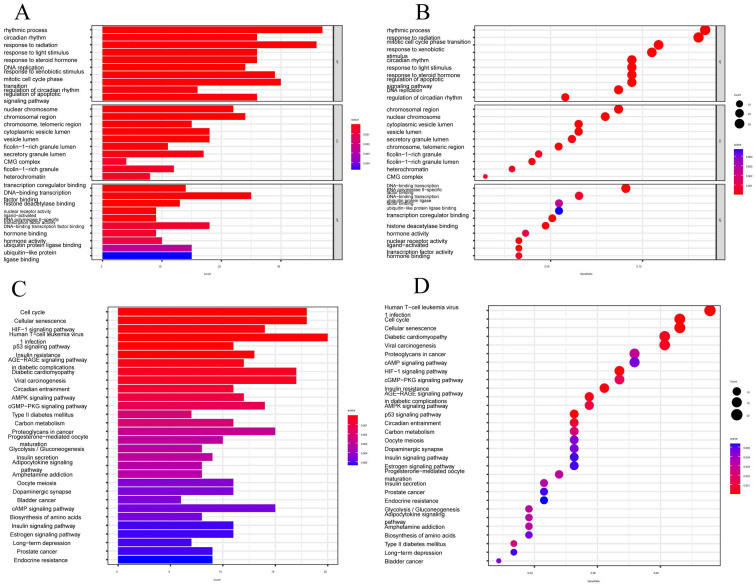
Functional enrichment analysis of CRGs. (A-B) The top 30 significant terms of GO, include MF, CC, and BP. (C-D) The top 30 significant pathways in KEGG enriched.

We performed GO functional analysis and Kyoto Encyclopedia of Genes and Genomes (KEGG) enrichment analysis to assess the differential gene expression in CESC, aiming to elucidate the interplay between biological activities, signaling pathways, and risk scores. The enrichment items were filtered using a significance threshold of p < 0.05, with a corrected p-value of <0.05 (FIGURE 5A-B). From the GO database, we identified 1615 relevant items, with a focus on biological processes (BP) such as lymphocyte and T-cell differentiation, as well as leukocyte adhesion, among others. Regarding cellular components (CC), the analysis encompassed various entities, including plasma membrane signaling receptor complexes, synaptic membrane constituents, and plasma membrane signaling receptor complexes. In terms of molecular functions (MF), the analysis highlighted receptor ligand activity, immune receptor activity, cytokine binding, and signaling receptor agonist activity. Furthermore, we identified the top 30 significant enrichment clusters derived from the GO functional analysis, which encompassed diverse processes such as circadian rhythm, regulation of the apoptotic signaling pathway, and rhythmic processes. In the context of KEGG enrichment analysis, the pathways primarily involved in CESC included the Cell Cycle, Cellular senescence, HIF-1 signaling pathway, and Human T-cell leukemia virus 1 infection (FIGURE 5C-D). These findings underscore the pivotal role of differentially expressed CRGs in the progression of CESC.

### CRGs risk score predicts TME and immune cell infiltration

The tumor microenvironment (TME) is composed of different cells that can influence the survival and metastasis of tumor cells (PMID: 34302814). In recent years, tumor therapeutic strategies targeting TME have demonstrated surprising potential (PMID: 33811125). It has been established that the interaction between cancer cells and the TME is critical for the development and spread of tumors 47. An essential part of the TME is TIICs, whose distribution and composition are intimately linked to tumor growth 46,48. Using the XCELL, TIMER, QUANTISEQ, MCPCOUNTER, CIBERSORT, CIBERSORT-ABS, and EPIC algorithms, we first investigated the relationship between risk ratings and the quantity of invading immune cells.

**Figure 6:**
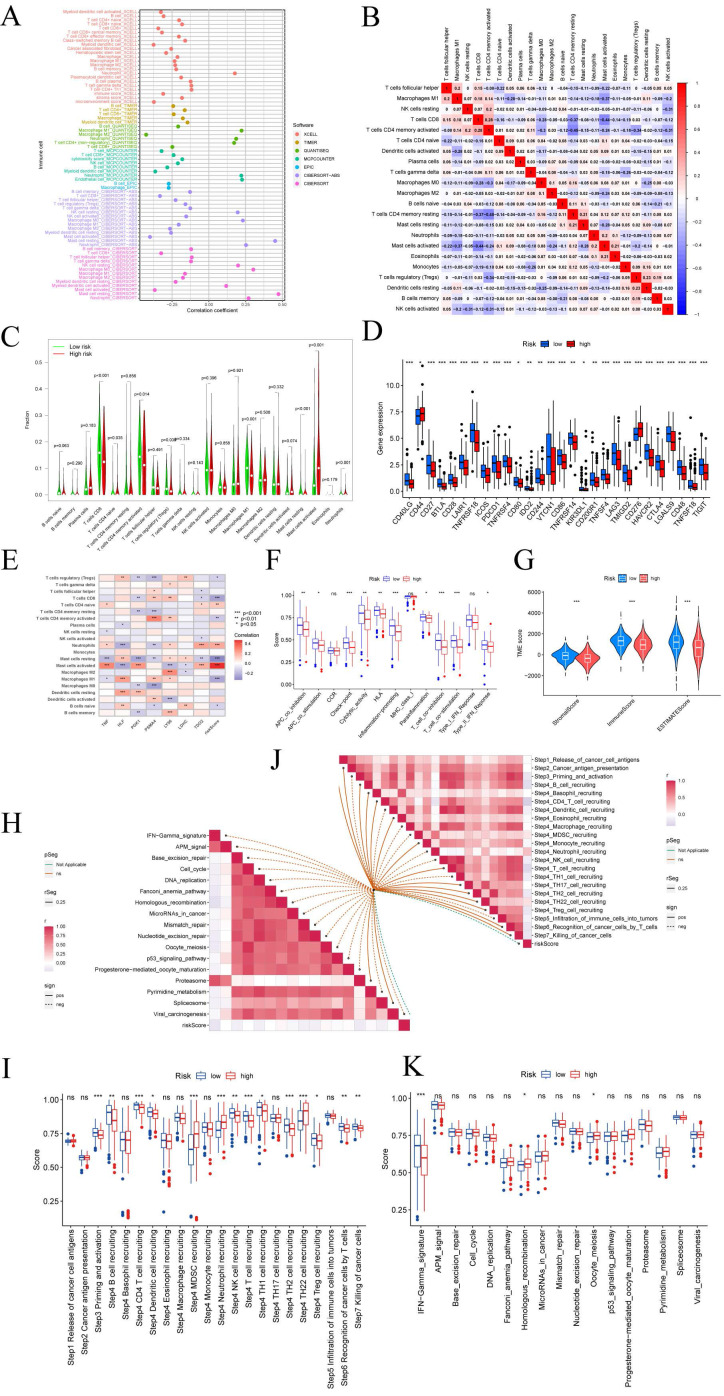
Prediction of CRGs risk scores for tumor microenvironment (TME) and immune cell infiltration. (A) Bubble plot of immune cells. (B) Correlation matrix of immune cells. Red indicates a positive correlation, blue indicates a negative correlation, and the color intensity represents the strength of the correlation. (C) Variations in immune cell infiltration between groups at high and low risk. (D) Immune checkpoint expression variations between high-risk and low-risk populations. (E) The relationship between immune cells and 7-CRGs. To determine correlation coefficients and p-values, Spearman correlation analysis was used. (F) Differences in immune cell and immunological function ssGSEA scores between groups at high and low risk. (G) Examination of TME subsystems. (H) Risk ratings and the ICB response signature have a positive correlation. (I) Correlation between each stage of the tumor immune cycle and risk ratings. (J) Disparities between high-risk and low-risk groups in the enrichment scores of immune treatment prediction pathways. (K) Differences between high-risk and low-risk groups at each stage of the cancer immune cycle. * P < 0.05; ** P < 0.01; *** P < 0.001.

The findings revealed an inverse relationship between risk scores and resting mast cells, neutrophils, and macrophage M1 (FIGURE 6A). The links between the various immune activities were shown by the correlation heatmap (FIGURE 6B). Immune cell infiltration is strongly associated with tumor therapy and patient prognosis. However, the role played by immune cell infiltration in CESC has not yet been investigated. So, using the CIBERSORT method, we independently assessed immune cell infiltration in CESC between the high-risk and low-risk groups. In contrast, the expression of T cells CD8, T cells CD4 memory activated, Macrophages M1, Mast cells resting, and Neutrophils was significantly higher in the low-risk group (FIGURE 6C). The expression of T cells CD4 naive, T cells regulatory (Tregs), and activated mast cells was significantly higher in the high-risk group. We examined the expression of immune checkpoint genes in the high-risk and low-risk groups in light of the significance of checkpoint-based immunotherapy.

Immune checkpoint genes CD44 and CD276 were found to be highly elevated in the high-risk group (FIGURE 6D), indicating that patients in this group may experience greater benefits from immune checkpoint blockade (ICB) therapy. Additionally, the risk score derived from our 7-CRGs model displayed a significant positive correlation with T cells CD4 naive, Neutrophils, and Mast cells activated, while it displayed a significant negative correlation with T cells regulatory (Tregs), T cells CD8, T cells CD4 memory activated, Mast cells resting, Macrophages M1, and B cells naive (FIGURE 6E). The infiltration of important immune cells was substantially correlated with the 7 CRGs, notably with HLF, PGK1, PSMA4, and LY96.

We examined the ssGSEA scores of immune functions because immune cells with checkpoint molecules can greatly affect immune activities. Several immune function scores in the high-risk group demonstrated significant variations when compared to the low-risk group (FIGURE 6F). Changes in the expression of immune cell types result in changes in the composition of the TME since infiltrating immune cells are a crucial component and characteristic of the TME. So, we looked at the TME components of the CESC samples. The results showed that the low-risk group's stromal score, immune score, and estimate score were all lower than those in the high-risk group, indicating greater immune levels and immunogenicity in the high-risk group's TME (FIGURE 6G).

We also examined the relationship between risk scores and ICB response characteristics in light of the crucial role that ICB response plays in immune checkpoint treatment (FIGURE 6H, J). It was discovered that there was a substantial positive link between homologous recombination and oocyte meiosis, but there was a significant negative correlation with the IFN-Gamma signature. A good association was found between risk scores and the immune cycle steps of MDSC recruitment, Neutrophil recruitment, and TH22 cell recruitment in the high-risk group (FIGURE 6I, K). We also performed correlation studies between risk scores and the phases of the tumor immune cycle.

### CRGs characteristics predict chemotherapy sensitivity

**Figure 7:**
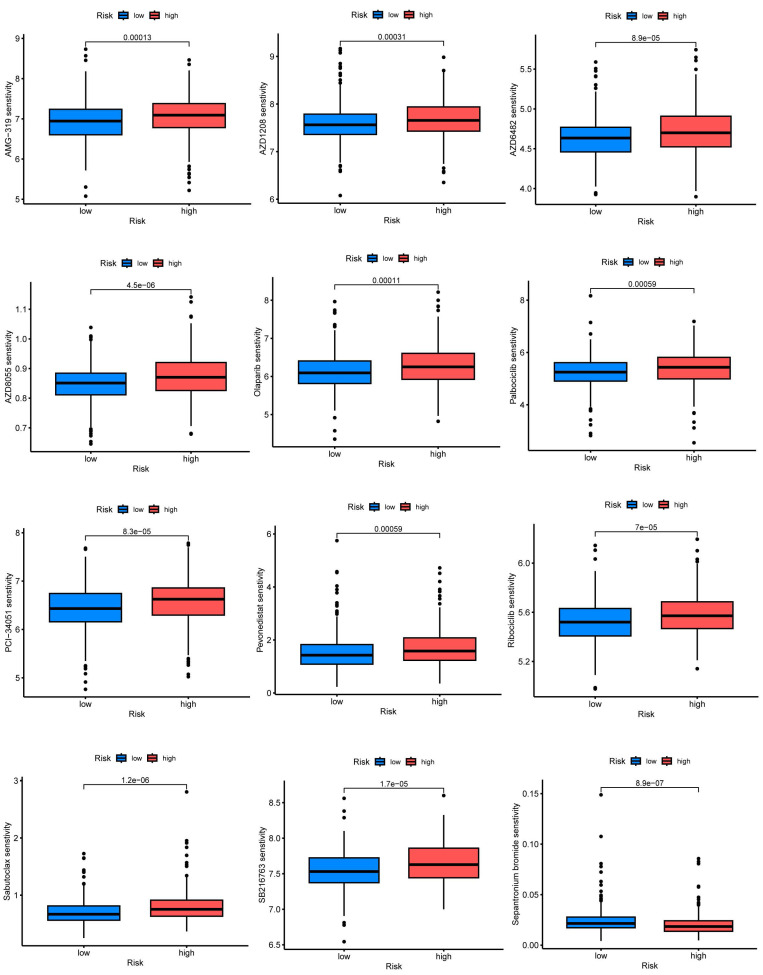
7-CRGs characteristics predict chemotherapy sensitivity. (A) AMG-319. (B) AZD1208. (C) AZD6482. (D) AZD8055. (E) Olaparib. (F) Palbociclib. (G) PCI-34051. (H) Pevonedistat. (I) Ribociclib. (J) Sabutoclax. (K) SB216763. (L) Sepantronium bromide.

Employing the risk score as a foundation, we conducted a comprehensive assessment of the prospective utility of 7-CRGs in prognosticating chemotherapy sensitivity within the GDSC database. Our objective was to augment the precision of pharmacological interventions. The dissimilarities in chemotherapy responsiveness between cohorts classified as high-risk and low-risk were meticulously scrutinized via utilization of the "pRophetic" R package, which facilitated a thorough exploration of this subject. Notably, a repertoire of twelve conventional inhibitors or medications was examined, thereby enriching our analysis. Within the high-risk group, discernible elevation in IC50 values was observed for AMG-319, AZD1208, AZD6482, AZD8055, Olaparib, Palbociclib, PCI-34051, Pevonedistat, Ribociclib, Sabutoclax, and SB216763(FIGURE 7A-K). Conversely, among the high-risk cohort, Sepantronium bromide demonstrated a reduced IC50 value, signifying a distinct pattern (FIGURE 7L). The integration of the risk score provides a framework for further exploration of immune therapy response in patients afflicted with CESC, thus bolstering the precision of pharmaceutical interventions and furnishing invaluable scientific insights for clinical investigations.

### The relevance of CRGs to the tumor microenvironment

We performed an in-depth analysis of the expression profiles of the 7-CRGs within the complex immunological microenvironment using the CESC_GSE168652 single-cell dataset obtained from the TISCH database.

**Figure 8:**
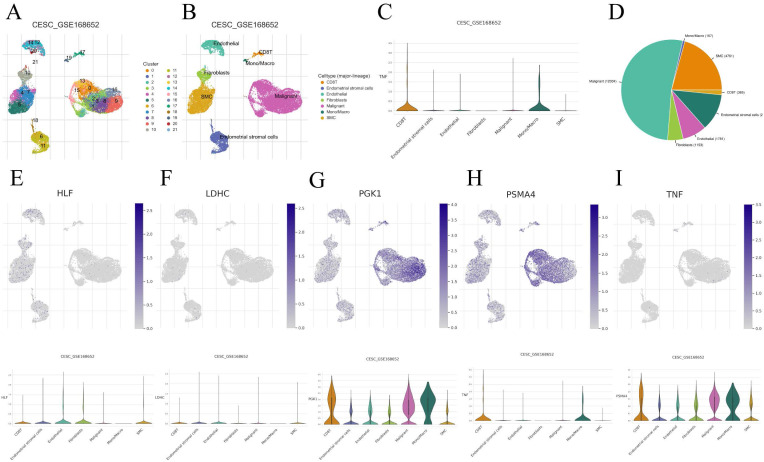
Correlation of CRGs with the tumor microenvironment. (A-D) Annotation of all cell types in CESC_GSE168652 and the percentage of each cell type. (E) HLF. (F) LDHC. (G) PGK1. (H) PSMA4. (I) TNF.

This comprehensive dataset encompasses a diverse range of 22 distinct cell clusters and encompasses 7 unique immune cell subtypes, facilitating a detailed depiction of the spatial distribution and relative abundance of each cell population (FIGURE 8A-D). Interestingly, the expression levels of the individual CRGs within the immunological microenvironment are notably absent in the HLF and LDHC populations (FIGURE 8E, F). In contrast, the expression of PGK1 and PSMA4 is observed across multiple immune cell subsets (FIGURE 8G, H), with TNF predominantly exhibiting expression within the Mono/Marco subpopulation (FIGURE 8I).

### Immunohistochemical staining and PCR confirm expression of biological clock gene proteins

**Figure 9:**
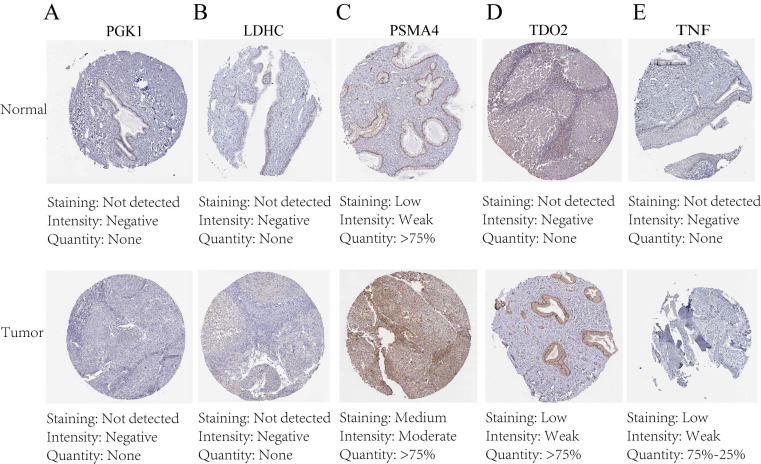
Immunohistochemical staining confirms the expression of the biological clock gene protein, Immunohistochemical analysis of 5 circadian clock genes in 7-CRGs. (A) PGK1. (B) LDHC. (C) PSMA4. (D) TDO2. (E)TNF.

The protein expression patterns of the five 7-CRGs we chose were subsequently examined in CESC tissues. PGK1 and LDHC protein expression levels were equivalent in carcinogenic and non-cancerous tissues, according to immunohistochemistry (IHC) analyses, showing no appreciable changes (FIGURE 9A-B). In contrast, it was discovered that CESC tissues had considerably higher levels of PSMA4, TDO2, and TNF expression (FIGURE 9C-E). The malignant development of CESC may be influenced by the overexpression of these genes. These findings offer new opportunities for immunotherapeutic approaches in CESC by validating the differential expression patterns of the 7-CRGs inside the immunological tissues of CESC patients, as predicted by our computational model.

## Discussion

CESC is the most common disease in the world and has a significant impact on women's health. Although early CESC has a favorable prognosis, the majority of cases are detected in the middle or late stages and have a less favorable prognosis. Therefore, the key to improving the prognosis of CESC is early diagnosis and monitoring. Finding new molecular markers is crucial for early diagnosis, determining prognosis, and improving the prognosis of CESC, particularly for the detection and treatment of advanced-stage malignancies.

Changes in the expression of circadian clock genes, whether they are up-regulated or down-regulated, contribute to tumor cell proliferation by upsetting the delicate balance of cell division 14, 49-52. The CRGs control a wide range of immunological activities in cancer, including immune infiltration, according to several lines of research 53-55. In solid tumors, CRGs control several immune procedures 56, 57. As an illustration, Kinker et al. 58 discovered a significant association between CESC and the proportions of various lymphocyte subsets, including CD4+ T cells and BK cells. Another separate group asserts that BMAL1 might function as a biomarker for T cell-based immunotherapies for melanoma 59.

The connection between the immunological infiltration in CESC and the circadian rhythm is still unclear, though. Therefore, analyzing tumor metastasis-related genes and establishing predictive models for tumor metastasis can provide an important basis for early intervention in CESC. However, there are not many known molecular markers that can be used in the clinical setting, and there is an urgent need to select molecular markers from these molecules that have high predictive power for the disease. The results of this project will provide an important theoretical basis for understanding the pathogenesis of the disease and finding new therapeutic approaches. we used multi-omics methods to investigate the relationship between the immune infiltration in CESC and the circadian clock.

In this study, we explored in depth the role of CRGs on the prognosis of CESC patients through machine learning techniques. By constructing a specific nomogram, we were able to effectively predict the prognosis of individual patients and identify circadian clock-related genes that may play other roles in CESC through techniques such as pathway enrichment analysis and immunoassay. Ultimately, the findings of this project will provide a theoretical basis for the clinical treatment of CESC and new ideas for the treatment of CESCs, which will further advance the development of CESC treatment.

First, we assessed the rhythm of 7 CRGs in Cervical squamous cell tissue. To assess the prognostic role of core biological clock gene expression in CESC, we used Kaplan-Meier survival analysis and Cox proportional risk regression analysis to assess the prognostic role of core biological clock gene expression in CESC. We found that core biological clock gene expression levels were associated with survival in CESC patients. To characterize the robust risk scores of seven genes as independent prognostic biomarkers, we identified TNF, HLF, PGK1, PSMA4, LY96, LDHC, and TDO2 as independent prognostic biomarkers. Previously investigators have described certain associations between independent prognostic biomarkers and cancer tumorigenesis and pathogenesis. For example, Balkwill, F demonstrated 60 that TNF-α is involved in pathological processes such as immune system maintenance and homeostasis, inflammation, host defense, chronic inflammation, and autoimmunity and that it is also involved in the development of malignant diseases. Similar to this, Hengyu Li et al. 61 discovered that TGF-1, a substance released by TAMs, controls HLF. Then, HLF promotes GGT1 to increase ferritin resistance, which fuels triple-negative breast cancer cell proliferation, metastasis, and cisplatin resistance. Furthermore, Yu He et al. 41 demonstrated that whereas high extracellular PGK1 expression suppresses cancer malignancy by stifling angiogenesis, high intracellular PGK1 expression promotes tumor cell proliferation. Additionally, PGK1 is linked to cancer patients' poor prognoses and resistance to chemotherapy and radiation therapy. According to Yushun Bai et al. 62, PSMA4 may influence lung cancer OS via cis-regulating the expression of the relevant genes.

LY96 was shown to be considerably increased by Kechao Nie et al. 63 in the majority of cancers, and their findings revealed that LY96 was connected to cancer copy number, DNA methylation, somatic mutation, microsatellite instability, tumor mutation load, and tumor size. Through the activation of the PI3K/Akt/GSK-3 pathway, Liangyuan Chen et al. 64 67 discovered that LDHC increased lung adenocarcinoma cell proliferation, migration, invasion, and epithelial-mesenchymal transition. By activating the Wnt5a pathway, TDO2 has been shown by Hui Liu et al. 65 to influence the expression of cancer-related biomarkers such as matrix metalloproteinase 7 (MMP7) and cell adhesion receptor CD44, which facilitates the migration and invasion of cancer cells. As a result, our data is consistent with earlier research on different cancers.

In light of this, categorizing samples based on already-created gene expression profiles is a useful strategy for solving this problem. Using this method, CRGs were used to categorize CESC patients. These genes' expression varied significantly across subtypes and was correlated with significantly different prognoses, indicating that our 7-CRGs markers could accurately predict a patient's prognosis. This makes it easier for clinicians to create various treatment plans. It demonstrates how the nomogram created based on the 7-CRG signature can assist physicians in creating more accurate, precise, and effective treatment plans that can benefit CESC patients at 1, 3, and 5 years.

The effectiveness of targeted therapies and the tumor metastatic process are both significantly influenced by the TME. We conducted several studies to learn more about this, and by examining the ratios of 22 immune cell types in various subtypes, we discovered that the levels of infiltration of T cells CD4 naive, T cells regulatory (Tregs), and Mast cells activated were significantly upregulated, indicating that they play a role in development. Additionally, each of the 7-CRGs was significantly linked to the invasion of important immune cells, including HLF, PGK1, PSMA4, and LY96, which are potential molecular targets for detecting and managing CESC. In conclusion, the nomogram based on the 7-CRGs model we created can assist doctors in creating individualized CESC treatment plans in clinical practice. It can also accurately predictt the survival of CESC patients. Future research on the molecular mechanisms underlying this trait will be clinically significant and may generate new concepts for precision medicine.

However, there are still some issues with this paper's study, particularly with our developed 7-CRGs model, which must be acknowledged and resolved in the subsequent research. First off, because our data analysis process is based on a public database, there may be differences between the results of our predictions and the real circumstances. We therefore tried to prevent this problem by obtaining additional data from CESC patients so that we could validate the model's usefulness and precision of immunotherapy predictions. Further experiments are needed to elucidate the underlying mechanisms of these characterized genes in this disease.

## Abbreviations

CESC: cell carcinoma and endocervical adenocarcinoma
CRGs: circadian clock-related genes
qPCR: quantitative real-time fluorescence PCR
SCC: squamous cell carcinoma
EAC: endocervical adenocarcinoma
HPV: high-risk human papillomavirus
FPKM: transcript per million mapped reads
TPM: transcripts per million
GSVA: gene set variation analysis
LASSO: least absolute shrinkage and selection operator
ROC: receiver operating characteristic
GO: Gene Ontology
IC50: inhibitory concentration
GDSC: Genomics of Drug Sensitivity in Cancer
TME: tumor microenvironment
TISCH: Tumor Immune Single Cell Hub
KM: Kaplan-Meier
OS: overall survival
TIICs: tumor immune infiltrating cells
SD: standard deviation
CDF: cumulative distribution function's
KEGG: Kyoto Encyclopedia of Genes and Genomes
BP: biological processes
CC: cellular components
MF: molecular functions
IHC: immunohistochemistry
